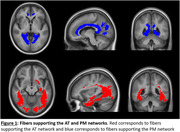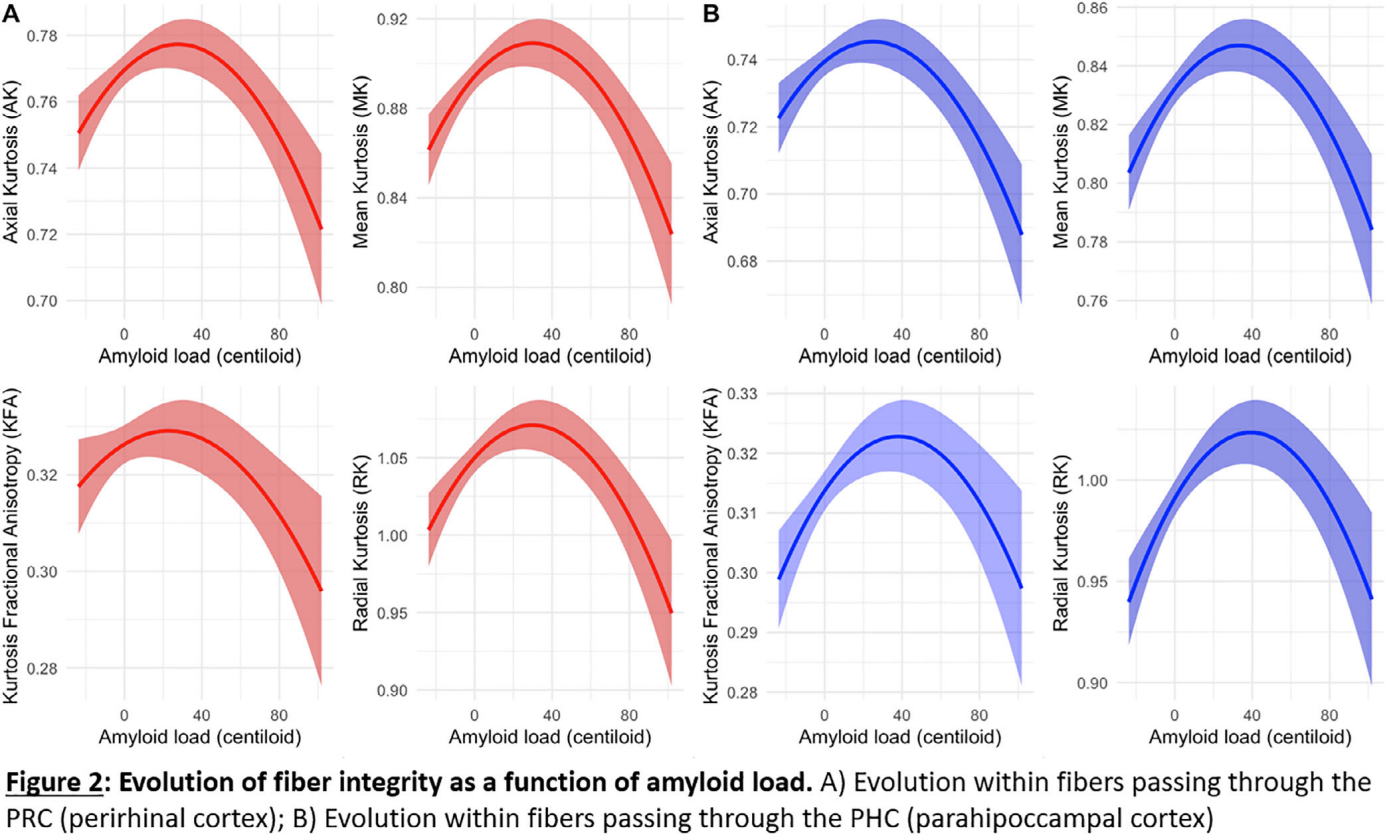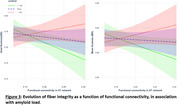# Structural connectivity of the medial temporal lobe: associations with amyloid load and functional connectivity

**DOI:** 10.1002/alz70856_098529

**Published:** 2025-12-24

**Authors:** Elise Saul, Jade Lasserve, Léa Chauveau, Brigitte Landeau, Géraldine Poisnel, Gael Chételat, Robin de Flores, the Medit‐Ageing Research Group

**Affiliations:** ^1^ Normandie Univ, UNICAEN, INSERM, U1237, PhIND “Physiopathology and Imaging of Neurological Disorders”, NeuroPresage Team, GIP Cyceron, Caen, France

## Abstract

**Background:**

Two networks within the medial temporal lobe (MTL)—the anterior‐temporal (AT) and posterior‐medial (PM)—are known to be functionally impaired in aging and Alzheimer's disease (AD). While our previous work suggested that AT hyperconnectivity is a pivotal mechanism in AD, and PM hypoconnectivity is canonical to aging, their structural connectivity is not fully understood. This study leverages longitudinal data to (i) identify the white matter fibers supporting the AT and PM networks, (ii) explore the effects of amyloid‐β (Aβ) accumulation on these fibers, and (iii) examine the link between fiber integrity and functional connectivity.

**Method:**

Eighty‐nine cognitively healthy older adults (68.96 ± 3.89 years) were included. Structural and functional connectivity within the AT and PM networks were assessed using resting‐state fMRI and DKI, respectively, using the perirhinal and parahippocampal cortices as seeds for correlation or tractography analyses. Amyloid‐β deposition was quantified with Florbetapir‐PET imaging.

**Result:**

The hippocampal cingulum and inferior longitudinal fasciculus were identified as independent components of both networks. Additionally, the AT network is supported by the thalamic radiations and corpus callosum, while the PM network is underpinned by the cingulum and inferior fronto‐occipital fasciculus. An inverted U‐shaped relationship was found between Aβ burden and white matter fiber integrity. Although no direct association was observed between fiber integrity and AT or PM functional connectivity, significant interactions with Aβ load were found: individuals with high Aβ levels showed a negative association between AT structural and functional connectivity, while those with low Aβ levels showed a positive correlation.

**Conclusion:**

These findings underscore the impact of Aβ on MTL structural connectivity and its association with increased AT functional connectivity, providing new insights into the complex relationship between connectivity and Aβ.